# Variant Profiling of a Large Cohort of 138 Chinese Families With Autosomal Dominant Retinitis Pigmentosa

**DOI:** 10.3389/fcell.2020.629994

**Published:** 2021-02-01

**Authors:** Ting Xiao, Yue Xie, Xin Zhang, Ke Xu, Xiaohui Zhang, Zi-Bing Jin, Yang Li

**Affiliations:** Beijing Ophthalmology & Visual Sciences Key Lab, Beijing Tongren Eye Center, Beijing Institute of Ophthalmology, Beijing Tongren Hospital, Capital Medical University, Beijing, China

**Keywords:** autosomal dominant retinitis pigmentosa, next-generation sequencing, diseasing-causing variant, copy number variation, variant profile

## Abstract

Retinitis pigmentosa (RP) is the most common form of inherited retinal dystrophy, and 15–25% of RP is transmitted as an autosomal dominant (ad) trait. The objectives of this study were to establish the variant profile in a large cohort of adRP families and to elucidate the variant spectrum of each adRP gene in Chinese patients. A total of 138 probands clinically diagnosed with RP as a presumed autosomal dominant trait were recruited. All probands underwent ophthalmic examinations by specialists. A combination of molecular screening methods, including targeted next-generation sequencing, Sanger DNA sequencing, and multiplex ligation probe amplification assay, was used to detect variants. We identified heterozygous variants of 11 adRP genes in 73 probands, hemizygous, or heterozygous variants of X-linked RP genes in six patients, compound heterozygous variants of autosomal recessive RP genes in three pseudodominant families, and one heterozygous variant of one ad cone and rod dystrophy gene in one proband. One proband was found carrying both variants in *RPGR* and *FAM161A*. The overall detection rate was 59.4% (82/138). We detected 72 distinct disease-causing variants involving 16 RP genes and one cone-rod dystrophy gene; 33 of these variants have not been reported previously. Disease-causing variants were identified in the adRP genes in 52.9% of the families, followed by 4.3% in the X-linked RP genes, and 2.2% in the autosomal recessive genes. The most frequent mutant genes were *RHO, PRPF31, RP1, SNRNP200*, and *PRPF8*, which explained up to 78.0% of the genetically diagnosed families. Most of the variants identified in adRP genes were missense, and copy number variations were common (7/20) in the *PRPF31* gene. We established the profile of the mutated genes and the variant spectrum of adRP genes in a large cohort of Chinese patients, providing essential information for genetic counseling and future development of therapeutics for retinal dystrophy inherited as a dominant trait.

## Introduction

Retinitis pigmentosa (RP) is the most common form of inherited retinal dystrophy (IRD), with a prevalence of about 1 in 4,000 (Ayuso and Millan, 2010). RP is a progressive disorder characterized by initial degeneration of rod photoreceptors, followed by degeneration of cone cells (Ayuso and Millan, 2010). Clinical features include night blindness (usually occurring in adolescence), progressive defects of the peripheral visual field, and ultimately, severe damage to central visual acuity (Ayuso and Millan, 2010; Daiger et al., 2014a). The typical fundus appearance consists of black bone-spicule pigmentation in the midperipheral retina, attenuated retinal arterioles, and a pale optic disc. Electroretinograms (ERGs) present reduced or non-recordable signals (Ayuso and Millan, 2010; Daiger et al., 2014a). Most RP cases (about 70–80%) are non-syndromic, which means that patients display only ocular dysfunction. However, some patients may present highly variable clinical symptoms and progression, even if they come from the same family (Ayuso and Millan, 2010).

RP is a highly genetically heterogeneous disorder and can be transmitted in an autosomal recessive (ar), an autosomal dominant (ad), or an X-linked recessive pattern (xl) (Ayuso and Millan, 2010; Daiger et al., 2014a). Digenic inheritance has been reported in some rare cases (Ayuso and Millan, 2010). Autosomal dominant RP (adRP) accounts for about 15–25% of the total RP cases (Daiger et al., 2014a). At present, 30 causative genes have been identified for adRP (RetNet: https://sph.uth.edu/retnet/home.htm), and more than 1,000 different kinds of variants have been described in those genes (Daiger et al., 2014a; Dias et al., 2018). The most commonly mutated gene is *RHO*, which is responsible for ~20–30% of adRP cases (Daiger et al., 2014a; Dias et al., 2018). Most of the detected variants in the adRP genes are private variants, and a small fraction of the common variants are ethnicity specific; for example, the *RHO* variant p.(P23H) is a founder variant almost exclusively described in Americans of European origin (Sullivan et al., 2013; Daiger et al., 2014a,b; Dias et al., 2018). Copy number variants (CNVs) have also been reported in some adRP genes, and incomplete penetrance has been observed in some specific genes (Sullivan et al., 2013; Daiger et al., 2014a,b; Dias et al., 2018). Taken together, these findings for adRP increase the complexity of its genetic diagnosis, which is very crucial for genetic consulting and gene therapy in patients with this disorder.

In recent years, the application of next-generation sequencing (NGS), mostly as targeted exome sequencing (TES) and whole exome sequencing (WES), has greatly increased the genetic diagnosis rates of different form of IRD (Daiger et al., 2014b; Xu et al., 2014; Costa et al., 2017; Van Cauwenbergh et al., 2017; Martin-Merida et al., 2018; Gao et al., 2019). The profile of mutated genes and the variant spectrum of adRP genes have been established in European and American patients with variant detection rates between 50 and 75% (Daiger et al., 2014b; Costa et al., 2017; Van Cauwenbergh et al., 2017; Martin-Merida et al., 2018). One very recent study that reported genetic analysis using TES in a large cohort of Chinese patients has shown that 72% of the patients could receive a molecular diagnosis (Gao et al., 2019). However, the large cohort included several other IRD cases, such as Bietti crystalline retinopathy, Leber congenital amaurosis, and retinitis punctata albescens, and it did not report the gene profiles for adRP patients (Gao et al., 2019). Several previous studies have reported the gene profiles in the relatively small Chinese adRP cohort (not more than 78 families) (Li et al., 2010; Xu et al., 2014; Huang et al., 2017).

In the current study, we have described the outcomes of a comprehensive molecular analysis of 138 pedigrees with possible adRP by a combination of methods, including TES, Sanger sequencing, and real-time quantitative polymerase chain reaction (q-PCR) analysis or multiplex ligation-dependent probe amplifications (MLPAs).

## Subjects and Methods

### Patients

In total, 138 unrelated families with a clinical diagnosis of RP were enrolled at the Genetics Laboratory of the Beijing Institute of Ophthalmology, Beijing Tongren Ophthalmic Center. Dominant inheritance was presumed when each family had affected members in at least two consecutive generations. This cohort included four previously reported families (Pan et al., 2012; Dong et al., 2013). Patients were diagnosed with RP based on the following criteria: a history of night blindness, progressive visual field defects, fundus displaying bone spicule-like pigment clumping in the midperipheral or peripheral retina and attenuation of retinal vessels, and severe rod-cone dysfunction or non-recordable ERG recording (Ayuso and Millan, 2010). The molecular testing processes were prospectively evaluated and approved by the ethics committee of Beijing Tongren Hospital, and all tests were implemented under the official guidelines of the Beijing Tongren Hospital Joint Committee on Clinical Investigation in compliance with the Declaration of Helsinki. Informed consent was obtained from probands after a detailed explanation of the processes.

Each proband and available family members underwent a regular ophthalmic examination that included best-corrected visual acuity, slit-lamp biomicroscopy, and a fundus examination. Most participants also underwent optical coherence tomography, visual field, and ERG evaluations. Peripheral blood samples were collected from patients and their family members, and genomic DNA was extracted from the leukocytes with a genomic DNA extraction and purification kit (vigorous whole-blood genomic DNA extraction kit; Vigorous Beijing, China), according to the producer's protocol.

### PCR-Based Sequencing of the *RHO* Gene

Five exons and flanking splicing sites of the *RHO* gene were first sequenced for all probands diagnosed with adRP, except for the four families that we have previously reported (Pan et al., 2012; Dong et al., 2013). Those four previously reported families were analyzed by linkage mapping following Sanger sequencing (Pan et al., 2012; Dong et al., 2013). The PCR amplifications were performed with regular reaction mixtures, and the purified amplicons were sequenced on an ABI Prism 373A DNA sequencer (Applied Biosystems, Foster City, CA, USA). The sequencing outcomes were matched to the available cDNA sequence of *RHO* (GenBank NM_000539).

### TES and Bioinformatics Analysis

We performed TES in 113 patients who did not carry any *RHO* variants using a capture panel developed and evaluated by our group (Sun et al., 2018). This panel comprised 188 known IRD genes, and 26 of them were adRP genes. Details of the procedures, which included the Illumina library preparation, capture experiments, and the enrichment libraries sequencing, have been described previously (Sun et al., 2018). The raw sequencing data processing, calling, and evaluation were carried out as previously reported (Sun et al., 2018). The bioinformatics programs PolyPhen2 (http://genetics.bwh.harvard.edu/pph/), Mutation Taster (http://www.mutationtaster.org/), and SIFT (http://sift.jcvi.org/) were used to predict the pathogenicity of each variant. The programs NetGene2 Server (http://www.cbs.dtu.dk/services/NetGene2/), Human Splice Finder (http://www.umd.be/HSF3/), and Berkeley Drosophila Genome Project (http://www.fruitfly.org/seq_tools/splice.html) were used to analyze any variants involving a splicing effect. We further determined the pathogenicity of each variant by searching the reported pathogenic variants in the HGMD database (http://www.hgmd.cf.ac.uk/ac/index.php) and the LOVD database (https://www.lovd.nl/). We ultimately classified the variants into pathogenic or likely pathogenic variants, variants of uncertain significance (VUS), and benign or likely benign variants according to the standards described by the American College of Medical Genetics and Genomics (ACMG) (Richards et al., 2015). Sanger sequencing was conducted to verify the supposed disease-causing variants and VUS. Segregation analysis was done for the probands and their family members.

### CNV Analysis and Validation

We employed the CNV kit software (https://github.com/etal/cnvkit) to identify CNVs from variations in the read depth for the patients who had TES data (Talevich et al., 2016). Real-time q-PCR was then performed to confirm the presence of the presumed CNVs of *PRPF31* in five families and of *FAM161A* in one family, as we previously reported (Dong et al., 2013). MLPA assays were conducted for three probands and their family members using the SALSA MLPA Kit P235 (Amsterdam, the Netherlands), following the producer's protocols.

### Supplementary PCR-Based Sequencing

We performed Sanger sequencing of exon 15 [open reading frame 15 (ORF15)] of *RPGR* in all male patients whose disease-causing variants were not found after TES and whose pedigrees could not exclude X-linked transmission.

### Statistical Analysis

All statistical analyses were performed using SPSS statistical software (version 25.0, SPSS Inc., Chicago, IL). The Kolmogorov–Smirnov test was used to evaluate whether the onset age of a single group conformed to a normal distribution. The Wilcoxon rank sum test was used to evaluate any difference in the onset age between two groups. *P* ≤ 0.05 was considered statistically significant.

## Results

### Variant Detection Rate and Variant Spectrum

**Figure 1:**
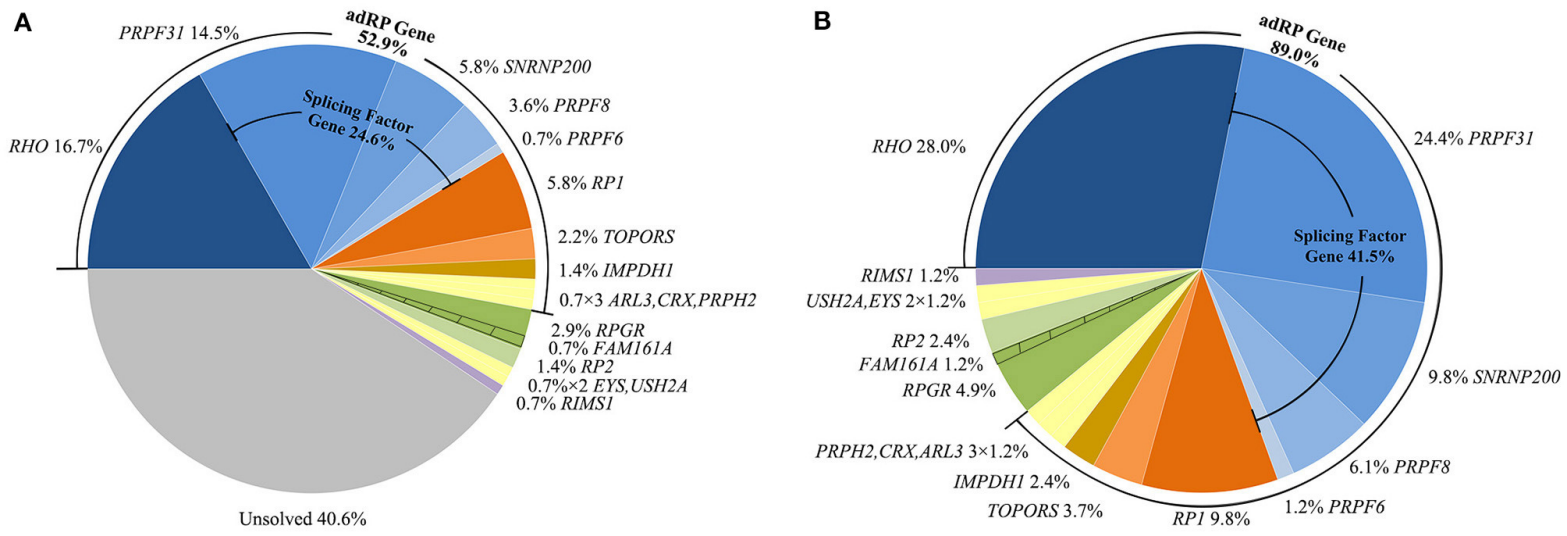
Summary of the proportion of patients in this study with variants in the involved genes. **(A)** Proportion of all probands with variants in each involved gene. **(B)** Proportion in all genetically diagnosed families with variants in each involved gene. The shaded areas indicate a proband carrying both variants of *RPGR* and *FAM161A*.

We identified heterozygous autosomal dominant variants that were pathogenic or likely pathogenic in 74 probands, hemizygous, or heterozygous in six probands, and compound heterozygous in three patients, for a general variant detection rate of 59.4% (82/138) (Table 1, Figure 1A). We found a total of 72 different variants in 17 genes, including 11 adRP genes, two xlRP genes, three arRP genes, and one autosomal dominant cone and rod dystrophy (adCORD) gene (Figure 1). Four of the 72 variants were identified three times or more, and the remaining 68 variants were detected either once (88.9%) or twice (5.6%). The most common variants were p.(R135W) and p.(P347L) in *RHO*, with a gene-specific allele frequency of 17.4% (4/23), followed by p.(R677^*^) in the *RP1* (37.5%, 3/8) and a whole *PRPF31* deletion (15.0%, 3/20) (Supplementary Table 1).

**Table 1:** Summary of Family Composition and Variant Screening Results in 138 Chinese adRP Families.

	**# Family with M to M**	**%**	**# Family without M to M**	**%**	**#Total**	**%**
adRP gene variant	40	64.5	33	43.4	73	52.9
adCORD gene variant	1	1.6	0	0.0	1	0.7
xlRP gene variant	0	0.0	6^*^	7.9	6^*^	4.3
arRP gene variant	2	3.2	1^*^	1.3	3^*^	2.2
No variant identified	19	30.7	37	48.7	56	40.6
Total	62		76		138	59.4

ad, autosomal dominant; ar, autosomal recessive; CORD, cone and rod dystrophy; M to M, male to male transmission; RP, retinitis pigmentosa; xl, x-linked;

* *one family carrying both variants of RPGR and FAM161A*.

**Figure 2:**
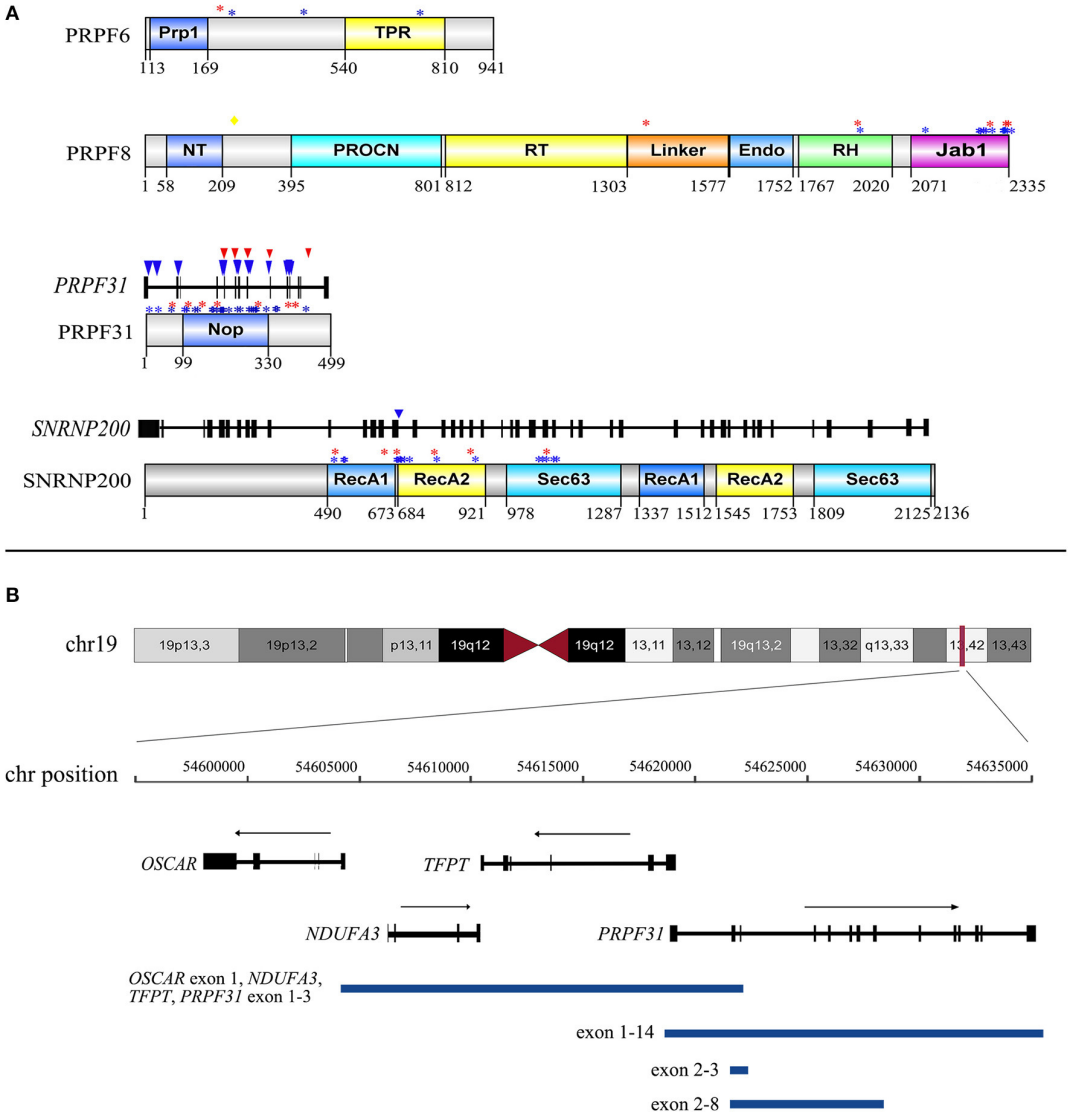
The distribution of 30 distinct variants of *PRPF31, SNRNP200, PRPF8*, and *PRPF6* identified in our study. **(A)** The distribution of 21 missense variants in the corresponding domain of *PRPF31, SNRNP200, PRPF8*, and *PRPF6* and the distribution of five splicing effect variants in the exons and introns of *PRPF31* and *SNRNP200*. Numbers under domains indicate amino acid location. Red asterisks indicate missense variants detected in the current cohort. Blue asterisks indicate missense variants reported in HGMD. Yellow diamond indicates the VUS of *PRPF8* detected in the current cohort. Red triangles indicate splicing effect variants detected in the current cohort. Blue triangles indicate splicing effect variants reported in HGMD. Prp1, pre-mRNA processing domain; TPR, tetratricopeptide repeat domain; NT, PRO8NT domain; RT, reverse transcriptase homology domain; Endo, restriction endonuclease homology domain; RH, RNase H homology domain; Jab1, Jun kinase activation binding protein; NOP, nucleolar protein domain; RecA1, repeat helicase ATP-binding domain; RecA2, repeat helicase C-terminal domain; Sec63, secretory-63 domain. **(B)** Lengths and positions of the four gross deletions involving *PRPF31* on chromosome 19q13.42.

Of the 72 putative disease-causing variants, 33 variants were first identified in the current study (Supplementary Table 1). These 33 novel variants comprised 16 missense, five frameshift indel, four non-sense, three splicing effect, three CNV, one synonymous, and one stop-codon-lost variant. None of these novel variants were recorded in our in-house and any public databases, such as the Exome Variant Server and 1000 Genomes Database (Supplementary Table 1). The 16 novel missense variants were defined as pathogenic or likely pathogenic variants according to the ACMG guidelines and standards (Supplementary Table 1). One synonymous variant c.1146G>A, p.(E382E), located in the last base of the exon 11 of *PRPF31*, was predicted to alter the downstream splice effect by NetGene2, HSF, and Mutation Taster. The remaining variants (frameshift small indel, non-sense, splicing effect, and run-on variants) or CNVs were considered to be obviously pathogenic variants. Five variants of *PRPF8* were identified in this cohort, and they all were novel. These five variants contained four missense variants and one frameshift small deletion that escaped non-sense-mediated decay. Three of these variants were located in the C-terminal Jab1 domain, and the other two were in the Linker and RNAseH-like domain (Figure 2A). We also detected an unreported missense variant, p.(S216G), which is not located in any domains of PRPF8 (Figure 2A). As p.(S216G) was predicted to be benign by both Polyphen2 and SIFT, we defined this variant as a VUS according to the ACMG guidelines and standards.

### Variant Profile in the adRP Genes and Related Clinical Features

The 59 putative disease-causing variants of the 11 adRP genes were identified in 73 pedigrees (52.9%, 73/138) and accounted for 89.0% (73/82) of the genetically diagnosed cases in this cohort (Figure 1B). Cosegregation analyses were performed in 65 pedigrees of the 73 families (Supplementary Table 2). The most frequently mutated gene was *RHO*, identified in 23 of the 138 probands (16.7%), followed by *PRPF31* in 20 unrelated patients (14.5%), *RP1* and *SNRNP200* each in 8 patients (5.8%), and *PRPF8* in 5 patients (3.6%) (Figure 1A). Variants in the pre-mRNA splicing factor genes accounted for 24.6% of the families and the variant locations in each gene are displayed in Figure 2A. Most of the variants in *RHO, SNRNP200, PRPF8*, and *IMPDH1* were missense variants, whereas loss-of-function variants, including non-sense, frameshift small indels, and CNVs, were more frequently observed in *PRPF31, RP1, CRX*, and *TOPORS* (Table 2). In the current cohort, CNVs were more prevalent in *PRPF31*, with a gene-specific frequency of 35.0% (7/20) (Figure 2B).

**Table 2:** Prevalence of Disease-Causing Variants in 138 Chinese adRP families and mutation type for each gene.

**Gene**	**No.Families**	**%**	**MS**	**FS**	**NS**	**SP**	**CNV**	**Others**
**adRP gene**								
*ARL3*	1	0.7	1	0	0	0	0	0
*CRX*	1	0.7	0	1	0	0	0	0
*IMPDH1*	2	1.4	2	0	0	0	0	0
*PRPF31*	20	14.5	3	1	2	5	7	2
*PRPF6*	1	0.7	1	0	0	0	0	0
*PRPF8*	5	3.6	4	1	0	0	0	0
*PRPH2*	1	0.7	0	1	0	0	0	0
*RHO*	23	16.7	20	0	3	0	0	0
*RP1*	8	5.8	1	3	4	0	0	0
*SNRNP200*	8	5.8	8	0	0	0	0	0
*TOPORS*	3	2.2	1	2	0	0	0	0
**xlRP gene**								
*RP2*	2	1.4	1	0	1	0	0	0
*RPGR*	4^*^	2.9	2	2	0	0	0	0
**arRP gene**								
*EYS*	1	0.7	1	0	1	0	0	0
*FAM161A*	1^*^	0.7	0	0	1	0	1	0
*USH2A*	1	0.7	2	0	0	0	0	0
**adCORD gene**								
*RIMS1*	1	0.7	1	0	0	0	0	0
**Not solved**	56	40.6						
**Total**	138	100						

ad, autosomal dominant; ar, autosomal recessive; CORD, cone and rod dystrophy; FS, frameshift; MS, missense; NS, non-sense; Other, run-on or synonymous variant; RP, retinitis pigmentosa; SP, splicing effect;

* *one proband carrying both variants of RPGR and FAM161A*.

**Figure 3:**
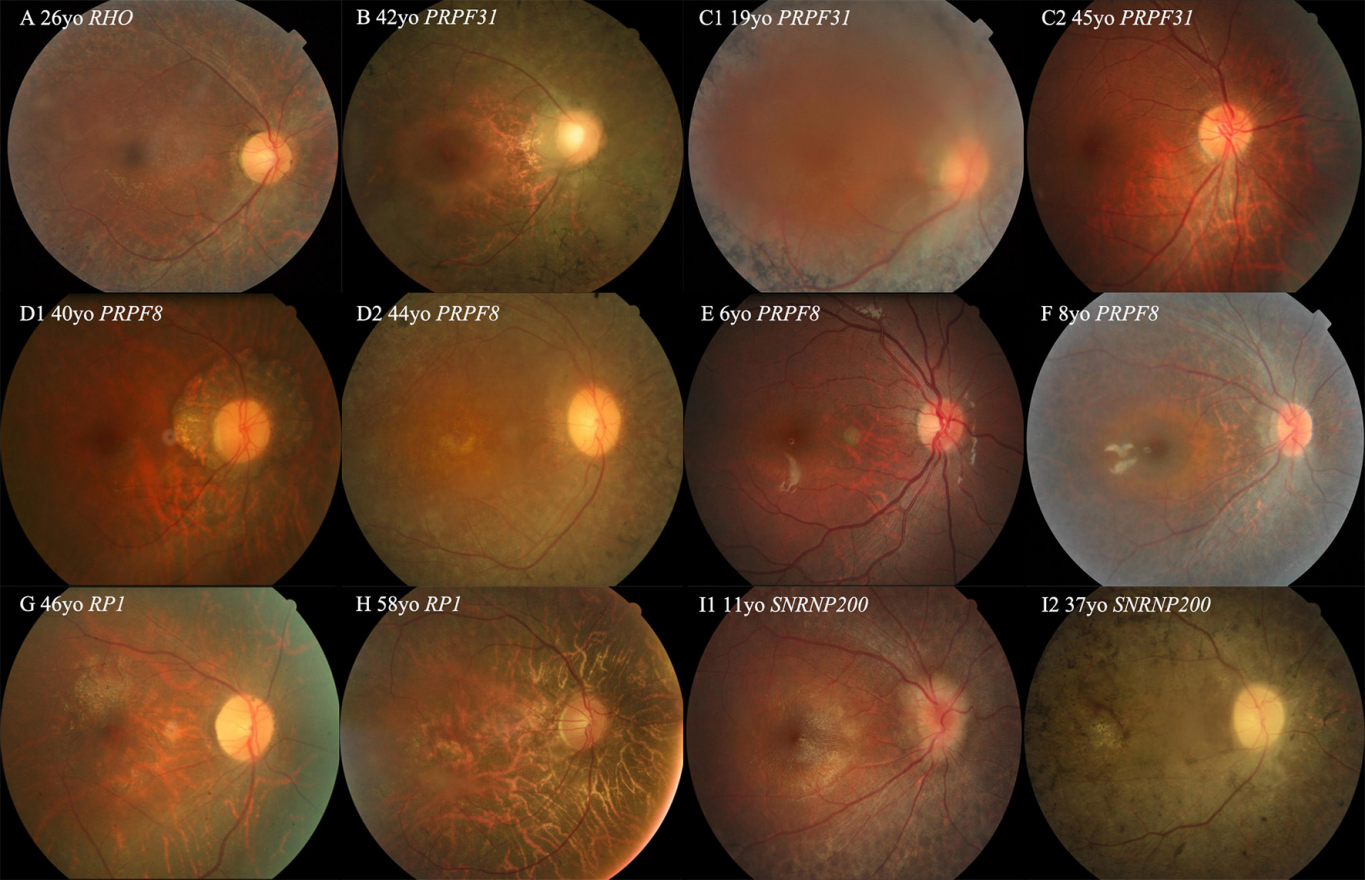
Colored fundus (CF) photographs of patients with variants of *RHO, PRPF31, SNRNP200, RP1*, and *PRPF8*. **(A)** CF image of proband 019875 with the missense variant of *RHO*. **(B)** CF photograph of proband 0191443 with exons 2–3 deletion of *PRPF31*. **(C1,C2)** CF photograph of proband 019417 with the non-sense variant of *PRRF31* shows dense black pigmentation in the midperipheral retina, but CF image of his 45-year-old father with the same variant displaying a normal fundus appearance **(D1,D2)** CF photographs of two affected sisters of pedigree 019917 with the missense variants of *PRPF8* located in the Jab1 domain. **(E,F)** CF photographs of two probands of 010215 and 019444 with the missense variants of *PRPF8* located in the Linker and RH region or domain. **(G,H)** CF photographs of probands 019545 and 0191212 with the variants of *RP1*. **(I1,I2)** CF images of two patients from pedigree 019756 with the missense variant of *SNRNP200*.

All probands with the adRP gene variants had typical RP symptoms, and their representative fundus appearances of the patients with variants in the first five common genes are shown in Figure 3. The median onset age of all patients with adRP genes was 5 years (range, 1–45 years) (Table 3). The median onset age was significantly older for the probands carrying the variants of *RP1* than for all probands with adRP (Table 3). Incomplete penetrance was observed in five pedigrees—three carrying variants in *PRPF31*, one in *PRPF8*, and one in *TOPORS*.

**Table 3:** Correlations between onset age of patients with different gene variants.

**Patients**	**Number**	**Onset age, years old**		
		**Mean ± SD**	**Median**	**Range**
Total adRP gene	73	10.7 ± 11.7	5	1–45
With *RHO* variants	23	7.5 ± 9.3	5	1–45
With *PRPF31* variants	20	6.5 ± 5.0	5	1–23
With *RP1* variants	8	33.1 ± 7.8	35^†^	20–41
With *SNRNP200* variants	8	5.1 ± 3.5	5	1–12
With *PRPF8* variants	5	6.6 ± 7.7	5	1–20
With *TOPORS* variants	3	10.0 ± 8.7	5	5–20
With other adRP gene variants	6	17.7 ± 16.6	9	5–40
With adRP gene variants except for *RP1* gene	65	7.9 ± 8.9	5^†^	1–45
With splicing factor gene variants (*PRPF31, PRPF6, PRPF8* and *SNRNP200*)	34	7.1 ± 7.4	5	1–38
Total xlRP	6	5.2 ± 0.4	5	5–6
With *RPGR* variants	4	5.5 ± 0.7	5.5	5–6
With *RP2* variants	2	5.0 ± 0	5	5

† *P = 0.000*.

### Variant Profiles in the xlRP and arRP Genes

**Figure 4:**
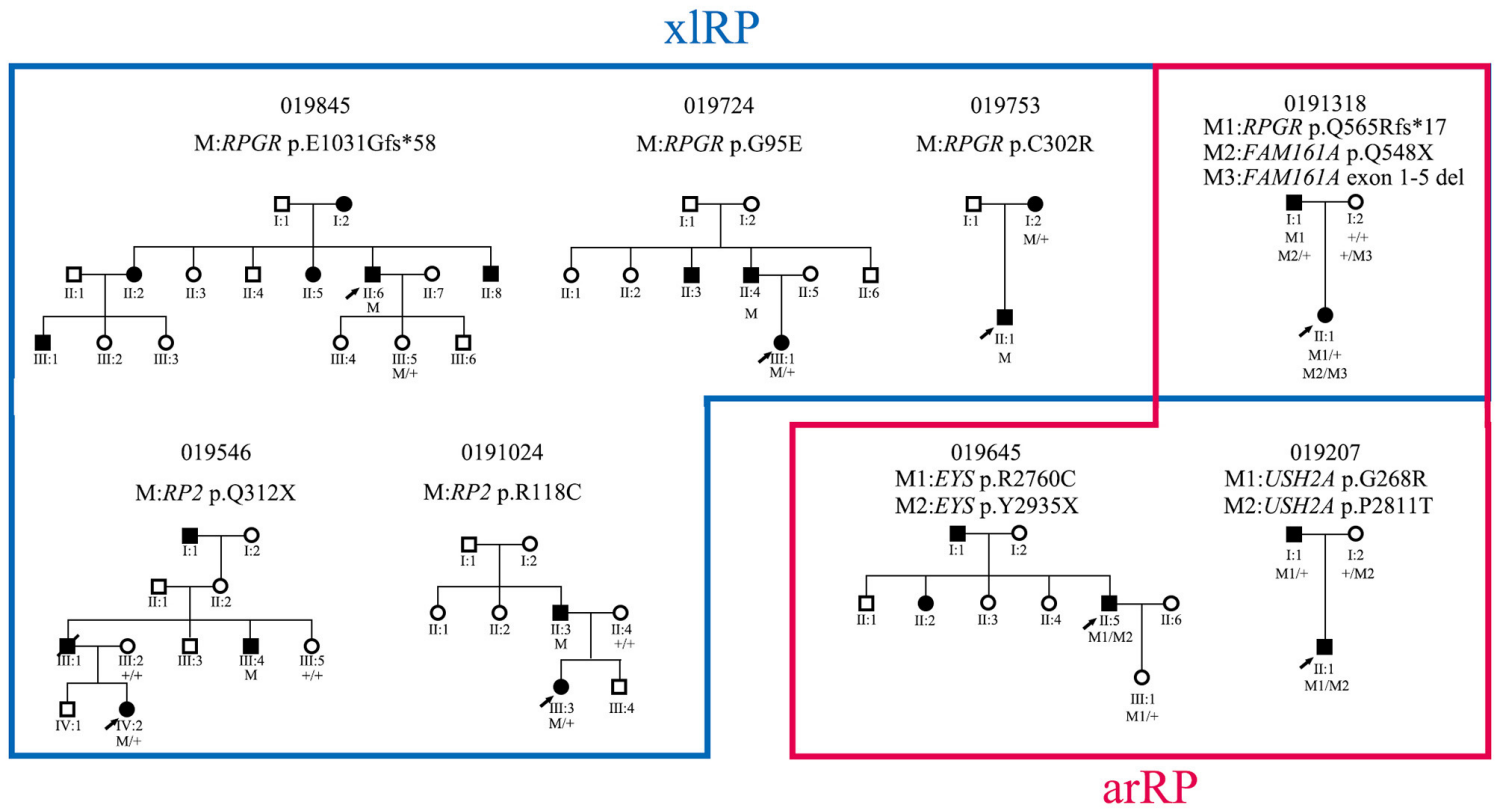
Pedigrees of eight families with xlRP and arRP gene variants and their cosegregation results.

Six hemizygous or heterozygous variants of the two xlRP genes were identified in six probands (4.3%, 6/138) and three compound heterozygous variants of three arRP genes in three probands (2.2%) (Figure 4). All six families with xlRP gene variants had at least two generations of inheritance; however, none of them showed male-to-male transmission that would exclude X-linked inheritance (Figure 4). Proband 0191318 was found to carry one heterozygous variant, p.(Q565Rfs^*^17), of *RPGR* and a compound heterozygous variant, M1:p.(Q548^*^) and M2:exon 1-5del, of *FAM161A*. Cosegregation analysis showed that his affected father carried a hemizygous variant of *RPGR* and a heterozygous variant, p.(Q548^*^), of *FAMI161A*. Pedigree 019207 was a small two-generation family with male-to-male transmission; however, the proband was found to harbor a compound heterozygous variant, p.[(G268R)]; [(P2811T)], of the *USH2A* gene. Cosegregation analysis showed that his affected father only carried a heterozygous variant, p.(G268R). We then conducted TES analysis on his affected father, but we did not detect any other disease-causing variant. Therefore, these two families were considered to be pseudodominant pedigrees (Figure 4). Proband 019645 was found to carry a compound heterozygous variant of *EYS*. During his family history review, he stated that his father and sister also had night blindness and visual acuity defects, but his father did not suffer from RP, according to a later ophthalmologic examination.

### Variants in Other IRD Genes

Proband 0191323 was found to carry a heterozygous variant, p.(S679T), of *RIMS1*, which is a disease-causing gene for adCORD. Cosegregation analysis showed that his affected mother also harbored this variant. The 51-year-old proband stated that he had experienced night-blindness since the age of 8 years and had developed obvious visual acuity loss and photophobia at around age 40 years. His fundus examination showed macular atrophy and black spicule-like pigment in the posterior pole and peripheral retina, and his ERG recording was extinguished. This patient might be in the late stage of CORD, so his fundus examination presented an RP-like appearance.

## Discussion

In the current study, we conducted a comprehensive molecular analysis in a large Chinese adRP cohort. We obtained an overall variant detection rate of 59.4% by means of several molecular methods, including linkage mapping, Sanger sequencing, targeted-exon sequencing, MLPA, and real-time q-PCR analysis. This detection rate is close to the rates recently reported in a large Spanish adRP cohort comprising 258 families (60%), as well as in a previously described small Belgian adRP cohort comprising 86 families (56%) (Van Cauwenbergh et al., 2017; Martin-Merida et al., 2018). However, it is much lower than the rate (70%) described previously in a large American adRP cohort that included 253 families (Daiger et al., 2014b). The variant detection rate is related to the accuracy of the probands' clinical diagnoses. In the current study, the solving rate was much higher for the families with male-to-male transmission (69.3%) than for the families without male-to-male transmission (51.3%). The variant detection rate is also related to molecular screening methods. For the large American adRP cohort, application of next-generation sequencing increased the detection rate from 65% (by only Sanger sequencing) to 70% (Daiger et al., 2014b).

Consistent with several previous studies, *RHO* was the most frequently mutated gene in the current adRP cohort, and its proportion (16.7%) was higher than the proportions reported in Chinese (8.9%) and Japanese (11.5%) adRP cohorts (Xu et al., 2014; Koyanagi et al., 2019) and close to those reported in French (18.3%), Italian (16.0%), and Belgian (14.0%) adRP cohorts (Ziviello et al., 2005; Coussa et al., 2015; Van Cauwenbergh et al., 2017), but much lower than those observed in American (26.9%) adRP cohorts (Daiger et al., 2014a,b). This difference might reflect, in part, the absence of the P23H variant, which is a founder variant and accounts for about 13.2% of the adRP in American families (Daiger et al., 2014a). The second most common mutated gene was *PRPF31*, which showed a 14.5% variant frequency in the current cohort. This frequency was higher than that observed in several adRP cohorts (6.8–10.5%) from Japan, Belgium, America, and Spain (Daiger et al., 2014a; Van Cauwenbergh et al., 2017; Martin-Merida et al., 2018; Koyanagi et al., 2019). The variant frequency of *SNRNP200* was also much higher in the current cohort than in the Spanish and American cohorts (5.8% vs. 2.3 or 1.5%) (Daiger et al., 2014a; Martin-Merida et al., 2018). Therefore, the findings that the variants in the pre-mRNA splicing factor genes were the major cause of the current Chinese adRP cohort and were responsible for 24.6% of the probands were not surprising (Figure 1B). By contrast, the variant frequency was much lower for *PRPH2* (0.7%) in our cohort than in the American, Belgian, and Spanish cohorts (7.0, 4.7, and 3.9%, respectively) (Daiger et al., 2014a; Van Cauwenbergh et al., 2017; Martin-Merida et al., 2018). The rate was also lower than the rate previously reported (3.7%, 3/79) in a small Chinese adRP cohort (Xu et al., 2014). Several previous studies have indicated that the variant frequency of *PRPH2* in adRP varies extensively with ethnicity, ranging from 0% (Mexican cohort) up to 10.3% (French cohort) (Sullivan et al., 2006; Matias-Florentino et al., 2009; Manes et al., 2015).

Our cohort showed a greater allelic diversity, and the majority of the observed variants (almost 90%) were private variants responsible for their own respective families. Only four variants in *RHO, PRPF31*, and *RP1* were detected three or more times. The most frequent variants, p.(R135W) and p.(P347L) in *RHO*, were variant hotspots that have been reported in several previous studies (Xu et al., 2014; Van Cauwenbergh et al., 2017; Martin-Merida et al., 2018). The common variant p.(R677^*^) in *RP1* was also a variant hotspot that has been described in many adRP families with different ethnicities (Bowne et al., 1999; Jacobson et al., 2000; Audo et al., 2012; Daiger et al., 2014a,b; Martin-Merida et al., 2018).

The proportion of novel variants identified in the current cohort was relatively high. In the current study, two novel missense variants, p.(R1384W) and p.(T1931M) of *PRPF8*, were not located in the C-terminal Jab1 domain (Figure 3A), where almost all reported variants are clustered (RuŽičková and Staněk, 2017). These two variants were situated in highly conserved regions and were predicted to be disease-causing by three *in silico* analysis programs and to cosegregate with their phenotype in the families. They were not found in any public databases, and we defined them as disease-causing variants according to the ACMG guidelines and standards. Additional functional studies are needed to verify their pathogenicity in the future.

Five distinct and large genomic DNA deletions of two genes were identified in eight probands. *PRPF31* showed a high prevalence (5.1%, 7/138) of CNVs in our adRP cohort; this prevalence was much higher than the prevalence of 1.9% recently reported in a large Spanish cohort (Martin-Merida et al., 2018). All five CNVs were in a heterozygous state; therefore, they were undetected by Sanger sequencing. Next-generation sequencing has the capability to detect CNVs, but this capability is related to the coverage depth. A previous study found that CNV analysis could generate ambiguous results in the target regions when the coverage was <250 × (Aparisi et al., 2014). In the current cohort, we used comprehensive screening methods, including linkage analysis in the early period, q-PCR, MLPA, and TES read count analysis, to detect the CNVs of *PRPF31*. This comprehensive analysis might be one of the reasons for the high CNV frequency for *PRPF31* observed in the present study.

Six probands (4.3%, 6/138) in the current cohort were found to harbor variants of the xlRP genes (*RPGR* and *RP2*). Four of the six probands were female and did not present a milder phenotype than was observed for the affected individuals in their families. Variant p.(E1031fs^*^58) (c.3092delA) located in the ORF15 of *RPGR* was identified in the supplementary PCR-based sequencing. The ORF15 where most *RPGR* variants are clustered is poorly covered in the NGS because of its highly repetitive sequence (Huang et al., 2015). Some variants in the ORF15 might be missed by NGS assays; therefore, alternative Sanger sequencing is necessary in patients from any adRP families without male-to-male transmission. A previous study using Sanger sequencing identified variants of *RPGR* and *RP2* in 8.5% (22/258) of adRP families (Churchill et al., 2013).

Obtaining a clear genotype–phenotype correlation is difficult because most probands harbor their own private variants in different genes. In the current cohort, we observed that patients with *RP1* variants had a late onset age and a mild phenotype when compared with patients with other adRP genes variants. This observation was consistent with previous descriptions (Jacobson et al., 2000; Audo et al., 2012). Incomplete penetrance was relatively common in the families with variants of *PRPF31*; however, its prevalence (15%, 3/20) was much lower than the 66.7% observed in Spanish patients (Martin-Merida et al., 2017). Three of our probands carried biallelic variants in three known arRP genes. Cosegregation analysis revealed pseudodominant inheritance in two families, with the remaining family unclear because of an incorrect family history report; this is one of the limitations—we did not perform ophthalmic evaluation in all affected family members of the current study.

Of the 56 probands whose disease-causing genes were not identified, 37 pedigrees did not show male-to-male transmission, and three families presented incomplete penetrance. In addition, 21 of the probands did not undergo ERG recording, which is another limitation of the current study as it means that the probability of a misdiagnosis could not be excluded. Their causal variants might be in areas not covered by our TES panel, such as the promoter or deep intronic regions of the targeted genes, or they may reside in newly identified genes that were not included in our TES panel. In the future, we will perform WES or WGS analyses for these patients.

In conclusion, we have established the profile of the mutated genes and the variant spectrum of adRP genes in a large cohort of Chinese patients, thereby providing essential information for genetic counseling and future therapeutic development for retinal dystrophy inherited as a dominant trait.

## Data Availability Statement

The original contributions presented in the study are included in the article/Supplementary Material, further inquiries can be directed to the corresponding author/s.

## Ethics Statement

The studies involving human participants were reviewed and approved by Ethics committee of Beijing Tongren Hospital. Written informed consent to participate in this study was provided by the participants' legal guardian/next of kin.

## Author Contributions

YL and Z-BJ designed and supervised the whole study. TX, YX, XinZ, KX, and XiaZ performed the experiments and interpret the results. TX wrote the manuscript. All authors contributed to the article and approved the submitted version.

## Conflict of Interest

The authors declare that the research was conducted in the absence of any commercial or financial relationships that could be construed as a potential conflict of interest.
